# Biodiversity and Food Security in Sub‐Saharan Africa: Evidence From a Dynamic Spatial Durbin Model

**DOI:** 10.1002/pei3.70232

**Published:** 2026-11-04

**Authors:** Etonam Julien Adangbedou, Boris Odilon Kounagbè Lokonon

**Affiliations:** ^1^ Laboratoire d’Economie Publique Université d’Abomey‐Calavi Abomey‐Calavi Benin; ^2^ Faculté des Sciences Economiques et de Gestion Université d’Abomey‐Calavi Abomey‐Calavi Benin; ^3^ Faculté des Sciences Economiques et de Gestion Université de Parakou Parakou Benin

**Keywords:** biodiversity, food security, Red List Index, Spatial Durbin Model, Sub‐Saharan Africa

## Abstract

This study examines the effect of biodiversity on food security in 40 Sub‐Saharan African countries over the period 2001–2022. Food security is measured using a composite Food Security Index constructed through Principal Component Analysis, while biodiversity is proxied by the Red List Index. To account for temporal persistence, spatial dependence and cross‐country spillover effects, the analysis employs a Dynamic Spatial Durbin Model. The results indicate that biodiversity has no statistically significant effect on the aggregate Food Security Index in either the short or the long run. However, disaggregated analyses reveal that biodiversity significantly improves Average Dietary Energy Supply Adequacy and the Share of Dietary Energy Supply Derived from Cereals, Roots and Tubers but has no significant effect on Average Protein Supply or Per Capita Food Supply Variability. Furthermore, robustness analysis using the prevalence of undernourishment demonstrates that biodiversity significantly reduces food deprivation, confirming that its contribution becomes more evident when food security is measured through specific nutritional outcomes rather than composite indicators. Among the control variables, food production and political stability improve food security, whereas unemployment and rapid population growth reduce it. The study underscores the need to integrate biodiversity conservation into agricultural and food security policies while promoting regional cooperation in ecosystem management across Sub‐Saharan Africa.

## 1 Introduction

Ensuring food security while conserving biodiversity has become one of the most pressing sustainability challenges of the 21st century (McKenzie and Williams 2015; Muluneh 2021; Nishigabiraki and Sekiyama 2025). This challenge is particularly acute in Sub‐Saharan Africa (SSA), where approximately one in four people remains undernourished and agriculture is simultaneously the primary source of livelihoods, the dominant land use, and a major driver of habitat loss (Clements et al. 2026; World Wildlife 2020). Consequently, understanding the relationship between biodiversity and food security is essential for achieving Sustainable Development Goals (SDGs) 2 (Zero Hunger) and 15 (Life on Land).

The relationship between biodiversity and food security has received increasing attention within the sustainability literature. The conceptual literature has organized the thoughts around the sustainability approach, which reads biodiversity as a form of natural capital whose yield is the flow of provisioning, regulating, supporting and cultural services that agriculture appropriates (Polasky and Daily 2021). Grounding on this framework, the ecosystem services approach identifies biodiversity as the biological foundation of provisioning, regulating, supporting and cultural services, including pollination, biological pest control, nutrient cycling, soil formation, water regulation and climate regulation, all of which sustain agricultural productivity and food security (Díaz et al. 2018; Millennium Ecosystem Assessment 2005). Collectively, these theoretical perspectives suggest that biodiversity is not merely an environmental concern but a fundamental determinant of sustainable food systems.

Empirical evidence generally supports the positive contribution of biodiversity to agricultural productivity and nutrition. Studies have shown that pollinator diversity enhances crop yields (Garibaldi et al. 2013; Woodcock et al. 2019), soil biodiversity improves ecosystem multifunctionality and agricultural productivity (Jiao et al. 2022; Wagg et al. 2014), biodiversity‐based pest control reduces crop losses (Dainese et al. 2019), and dietary biodiversity strengthens nutrient adequacy (Lachat et al. 2018). Furthermore, biodiversity contributes more strongly to ecosystem functioning under environmental stress, highlighting its importance for climate‐resilient agriculture (Hong et al. 2022). However, other studies report that biodiversity has only a limited direct effect on aggregate food security after controlling for socioeconomic factors such as income, agricultural technology, infrastructure and institutional quality (Musa et al. 2025; Vu et al. 2026). Some authors also argue that biodiversity conservation may constrain agricultural expansion by limiting access to productive land, particularly in regions experiencing rapid population growth (Song et al. 2025). This debate is reflected in the long‐standing discussion between land‐sharing and land‐sparing strategies as alternative approaches to balancing agricultural production and biodiversity conservation (Kremen 2015; Phalan et al. 2011).

Despite these advances, important gaps remain in the literature. First, most studies rely on farm‐level experiments or local case studies, limiting the generalizability of their findings to national or regional scales. Second, biodiversity is often represented by narrow indicators such as pollinator abundance or forest cover, which capture only specific aspects of biodiversity. In contrast, the Red List Index (RLI) provides a comprehensive measure by tracking changes in species extinction risk using standardized IUCN criteria (Butchart et al. 2004; IUCN 2022). Third, existing studies largely overlook the spatial nature of biodiversity and ecosystem services. Since ecosystems, wildlife populations, watersheds, and climatic processes frequently cross national boundaries, biodiversity changes in one country may influence food security in neighboring countries through ecological and economic spillover effects. Ignoring these spatial interactions may therefore produce biased estimates of the biodiversity–food security relationship.

These gaps are particularly relevant for SSA, which contains globally significant biodiversity hotspots while simultaneously experiencing persistent food insecurity, rapid population growth, agricultural expansion, deforestation, land degradation and increasing climate change (FAO, IFAD, UNICEF, WFP, WHO 2023). Understanding whether biodiversity contributes to food security under these conditions is essential for designing policies that simultaneously promote environmental sustainability and food security.

This study contributes to the literature in three important ways. First, it examines both a composite Food Security Index (FSI) and its individual dimensions, complemented by robustness analysis using the prevalence of undernourishment to assess the biodiversity–food security nexus. Second, it explicitly accounts for spatial dependence by estimating a Dynamic Spatial Durbin Model (SDM), thereby capturing both direct effects and cross‐border spillover effects of biodiversity on food security. Third, by combining dynamic panel methods with spatial econometrics, the study addresses temporal persistence, endogeneity and spatial interactions, providing more robust evidence on the biodiversity–food security nexus in SSA.

Against this background, the study seeks to answer the following research question: Does biodiversity improve food security in SSA, and to what extent do biodiversity spillovers across neighboring countries influence food security outcomes? Accordingly, the main objective is to examine the effect of biodiversity on food security in SSA while accounting for dynamic adjustment and spatial dependence.

The remainder of the paper is organized as follows. Section 2 reviews the theoretical and empirical literature on biodiversity and food security. Section 3 presents the methodological framework, including the theoretical model, empirical specification and estimation strategy. Section 4 reports and discusses the empirical findings. Finally, Section 5 concludes the paper and highlights the main policy implications.

## 2 Literature Review

Food security, in the working definition adopted by the global policy community and reproduced in the *Living Planet Report 2020*, exists ‘when all people, at all times, have physical, social and economic access to sufficient, safe and nutritious food that meets their dietary needs and food preferences for an active and healthy life’ (World Wildlife 2020). That definition already implies an analytical problem: food security is a multidimensional condition that cannot be reduced to caloric supply, and its determinants span ecosystems, markets and institutions.

### 2.1 Theoretical Traditions Linking Biodiversity to Food Security

Four theoretical traditions dominate the conceptual literature. The first is the ecosystem‐services tradition descending from the *Millennium Ecosystem Assessment* (MEA) and continuing through *The Economics of Ecosystems and Biodiversity* and the Intergovernmental Science‐Policy Platform on Biodiversity and Ecosystem Services (IPBES) (Dawson et al. 2019). This tradition frames biodiversity as the biological substrate from which services flow and offers the supply‐side accounting grammar by which regulating and supporting services such as pollination, pest control, soil formation, water purification, enter food‐system analysis alongside provisioning services such as food, feed, fiber and genetic resources (Scherr and McNeely 2008).

A second tradition centers on agrobiodiversity and the diversity–stability hypothesis. Drawing on the *experimental ecology* of Tilman and Downing (1994) and the *bioeconomic reconceptualization of resilience* by Perrings (1995), environmental economists have argued that genetic variation within species, and functional and response diversity across species, raise the productive capacity and the buffering capacity of agricultural systems against environmental perturbations (Di Falco and Chavas 2008). Walker et al. (2006) formalized the conceptual core in two distinct forms of diversity: functional diversity (the number of functionally different groups influencing system performance) and response diversity (the diversity of responses to disturbance within a functional group, influencing resilience).

The third tradition addresses *nutritional diversity*, linking consumption‐side food security to biological variation in production (Johns 2003; Lachat et al. 2018). Lachat et al. (2018) operationalized ‘dietary species richness’ as the most defensible metric of food biodiversity in diets and demonstrated, using 24‐h dietary recall data from 6226 participants across seven low‐ and middle‐income countries, that for every additional species consumed, dietary nutrient adequacy rose by 0.03, and that combining maximum species richness with maximum diet diversity score most reliably predicted micronutrient adequacy. Johns (2003) had earlier argued, from a complementary register, that dietary diversity supplies an intrinsic buffer against uncertainty in optimal diet specifications and against environmental change, and that simplification of diets across the nutrition transition constitutes a public health externality of biodiversity loss.

A fourth tradition is the sustainable livelihoods framework, which situates biodiversity for food and agriculture within a household asset portfolio of natural, physical, financial, social and human capital, framing rural households as combining these capitals to manage a vulnerability context (Dawson et al. 2019). In this register, biodiversity underwrites not only directly consumable provisioning services but also the resilience of coping strategies, including diversified wild harvesting, semi‐domesticated species and non‐market exchange.

### 2.2 Biodiversity and Food Security: An Empirical Review

Pollination is one of the most extensively studied mechanisms linking biodiversity to food security. Animal pollination supports the reproduction of approximately 87% of flowering plant species worldwide and plays a critical role in sustaining agricultural productivity and nutritional security (Hanley et al. 2015). Empirical studies consistently show that pollinator decline reduces crop yields, threatens the production of micronutrient‐rich foods and generates substantial economic losses (Lippert et al. 2021; Potts et al. 2016). Estimates suggest that a complete collapse of pollination services could reduce global welfare by 1%–2% of global GDP, underscoring the economic importance of pollinator conservation (Lippert et al. 2021).

A large body of empirical evidence demonstrates that both managed honey bees and wild pollinators contribute significantly to crop production, although wild pollinators often provide complementary services that cannot be fully replaced by managed bees (Garibaldi et al. 2013; Reilly et al. 2024). Moreover, greater pollinator diversity and abundance consistently enhance crop yields, reduce yield gaps, and improve crop quality across a wide range of agricultural systems (Garibaldi et al. 2016; Gazzea et al. 2023; Woodcock et al. 2019). These findings indicate that biodiversity contributes to food security not only by increasing agricultural output but also by improving the nutritional quality of food.

However, growing evidence also points to a widespread decline in pollinator populations driven by agricultural intensification, urbanization and habitat degradation (Millard et al. 2021; Powney et al. 2019; Raven and Wagner 2021). These declines threaten the long‐term sustainability of agricultural production and reinforce the need for biodiversity conservation policies that protect pollinator diversity as a means of enhancing food security.

Biodiversity‐based pest control is also an important ecosystem service that enhances agricultural productivity by increasing the abundance and diversity of natural enemies of crop pests. Empirical evidence consistently demonstrates that diverse predator and parasitoid communities suppress pest populations more effectively than simplified agricultural systems, particularly in landscapes with greater habitat complexity (Dainese et al. 2017). Studies further show that higher species diversity, community evenness, and stronger ecological interactions significantly reduce pest abundance, thereby lowering crop losses and reducing reliance on chemical pesticides (Lundgren and Fausti 2015).

The effectiveness of biological pest control depends on both landscape characteristics and pest life‐history traits, with conservation of non‐crop habitats enhancing the complementary roles of different natural enemy species (Duru et al. 2015; Wilby and Thomas 2002). Recent meta‐analyses confirm that biodiversity improves pest regulation across a wide range of cropping systems, while simplified agricultural landscapes often experience higher pest outbreaks due to reduced populations of beneficial insects (Cortez‐Madrigal and Gutiérrez‐Cárdenas 2023). These findings highlight biodiversity as a cost‐effective natural pest management strategy that supports sustainable agricultural production, reduces dependence on synthetic pesticides and ultimately contributes to improved food security.

A growing body of empirical evidence demonstrates that biodiversity enhances ecosystem multifunctionality and strengthens the resilience of agricultural systems to climate change and environmental stress. Studies consistently show that biodiversity contributes more strongly to ecosystem functioning when multiple ecosystem services are considered and that its positive effects become even greater under adverse climatic conditions (Hong et al. 2022; Lefcheck et al. 2015). Moreover, interactions between above‐ and belowground biodiversity improve ecosystem multifunctionality, with climate acting as an important mediator (Jing et al. 2015). These findings support the natural capital perspective, which views biodiversity as an asset whose value increases during periods of environmental stress when food insecurity is most severe (Dasgupta and Levin 2023). Empirical evidence further indicates that biodiversity enhances agricultural resilience by increasing productivity under unfavorable rainfall conditions, thereby buffering food systems against climate variability (Di Falco and Chavas 2008).

## 3 Data and Methodology

### 3.1 Data

#### 3.1.1 Measurement of Biodiversity

Biodiversity is measured using the RLI, which is developed by the International Union for Conservation of Nature (IUCN). The RLI tracks changes in the aggregate extinction risk of species over time and reflects the overall condition of biodiversity. The index ranges from 0 to 1, where values closer to 1 indicate better biodiversity quality and a lower risk of species extinction, while lower values signify greater biodiversity degradation and increased extinction risk. The RLI is widely used in environmental and sustainability research (Martínez and Melo 2025; Rogers 2025; Smith et al. 1993). It provides a comprehensive measure of changes in species conservation status and is one of the official indicators for monitoring progress towards the SDGs, particularly SDG 15 (Life on Land).

The RLI is available for five taxonomic groups: birds, mammals, amphibians, cycads and warm‐water reef‐building corals. To calculate the RLI for individual countries or regions, each species contributing to the index is weighted by the proportion of its global range in the country or region in question, based on the methodology published by Rodrigues et al. (2014). The resulting index therefore shows the overall risk of species extinction in the country or region in relation to its potential contribution to global species extinction risk. For more details on how the RLI is calculated, see Butchart et al. (2005).

#### 3.1.2 Construction of the Composite FSI

The dependent variable used in this study is the Composite FSI. Following the approach proposed by Jolliffe (2002), the index was constructed using Principal Component Analysis (PCA) (Table A.1). The FSI was derived from four indicators commonly used to capture key dimensions of food security: (i) Average Dietary Energy Supply Adequacy (ADESA), (ii) Average Protein Supply (APS), (iii) the share of dietary energy supply obtained from cereals, roots, and tubers (SDES), and (iv) per capita food supply variability (PCFSV) (Gnedeka et al. 2026; Leser 2013).

The number of components retained was determined using Kaiser's criterion (Kaiser 1974), which recommends retaining only components with eigenvalues greater than one. The results indicate that the first and second principal components have eigenvalues of 1.855 and 1.100, respectively, explaining 46.37% and 27.51% of the total variation across the four indicators (Table A.1). Since the remaining components had eigenvalues below the threshold of one, the first and second principal components were retained and used to construct the FSI.

Although the Kaiser–Meyer–Olkin (KMO) measure of sampling adequacy is relatively modest (0.448 < 0.5) (Table A.2), the use of PCA remains appropriate in this context. As noted by Booysen (2002), multidimensional concepts such as food security often comprise indicators that are conceptually related but not necessarily highly correlated. Consequently, moderate KMO values do not invalidate the construction of composite indices when the selected variables represent distinct yet complementary dimensions of the underlying concept. Moreover, Jolliffe (2002) argues that PCA is suitable for constructing composite development indicators provided that the retained components capture a substantial proportion of the total variation. Since the first two principal components explain more than 50% of the total variance, the resulting FSI provides a parsimonious and theoretically grounded measure of food security for the empirical analysis.

#### 3.1.3 Description of the Variables

We use data for 40 sub‐Saharan African countries covering the period 2001–2022. The adopted time frame and scope are based on data availability constraints. The selected variables are based on the literature.

Table 1 below presents the description and the sources of the variables used.

**TABLE 1:** Description of the variables.

Variables	Description	Sources
*Dependent variable*		
Food Security Index (FSI)	Composite index constructed using Principal Component Analysis (PCA)	Author's computation based on FAOSTAT (https://www.fao.org/faostat/en/#data/FS) (2025)
*Independent variables*		
Red List Index (RLI)	Measures biodiversity quality by tracking changes in species' extinction risk over time.	IUCN Red List Database (https://www.iucnredlist.org) (2025)
*Control variables*		
Food Production Index	Measures changes in the aggregate volume of agricultural production. Higher values indicate greater agricultural output.	WDI World Bank (https://databank.worldbank.org/source/world‐development‐indicators) (2025)
Political stability	Measuring perceptions of political stability and the likelihood of political unrest. Higher values indicate greater political stability	WGI World Bank (https://www.worldbank.org/en/publication/worldwide‐governance‐indicators)
Unemployment rate	Percentage of the total labor force that is unemployed but actively seeking employment	WDI World Bank (2025)
Population	Total resident population of a country	WDI World Bank (2025)
Gross domestic product per capita	Total income earned through the production of goods and services in an economic territory divided by the general population	WDI World Bank (2025)

*Source:* Authors compilation (2026).

### 3.2 Methodology

#### 3.2.1 Theoretical Framework

The theoretical foundation originates from the classical Malthusian theory of food security developed by Malthus (1798). Malthus argued that while food production expands only arithmetically, population grows geometrically, eventually creating pressure on food availability and increasing the likelihood of food insecurity. Under this framework, food security is fundamentally determined by the balance between agricultural production and population growth. When population growth exceeds the productive capacity of agriculture, food shortages emerge unless technological progress or productivity improvements shift the production frontier outward. Consequently, food security can be viewed as a function of agricultural production and demographic pressure,

(1) $$\mathrm{FSI}=f\left(\mathrm{AP},\mathrm{POP}\right)$$

where FSI denotes food security, AP represents agricultural production and POP denotes population.

However, modern food systems extend beyond the Malthusian perspective because agricultural production itself depends upon multiple productive assets.

The neoclassical production theory explains agricultural output as the result of combining productive inputs such as capital K, labor L, Natural conditions N and technology T (Rosine and Helmberger 1974). Following the Cobb–Douglas production framework,

(2) $$Y=\mathrm{AP}=f\left(K,L,N,T\right)$$

Traditional agricultural economics often treats land as the principal natural resource. However, environmental economics increasingly recognizes, through the Natural Capital Framework, that land quality depends critically upon biodiversity and ecosystem services (Dasgupta 2021; Dasgupta and Levin 2023). Recent empirical studies demonstrate that biodiversity improves crop productivity through pollination (Reilly et al. 2024), enhances soil fertility (Jiao et al. 2022), strengthens biological pest control (Dainese et al. 2017), and increases resilience against climate shocks (Hong et al. 2022).

Consequently, agricultural production may be rewritten as follows:

(3) $$\mathrm{AP}=f\left(K,L,N,T,B\right)$$

where B denotes biodiversity.

Substituting this production function into the Malthusian food security relationship yields

(4) $$\mathrm{FSI}=f\left(K,L,N,T,B,\mathrm{POP}\right)$$

This expression establishes biodiversity as a determinant of food security through agricultural production.

Food security is also influenced by socioeconomic conditions. The Sustainable Livelihoods Framework (Dawson et al. 2019; Scoones 1998) views household welfare as depending upon five forms of capital: natural, human, financial, physical and social capital.

Therefore, we incorporate these variables into the model as control variables. The model becomes:

(5) $$\mathrm{FSI}=f\left(K,L,N,T,B,\mathrm{POP},\mathrm{FPI},\mathrm{PS},\text{UNEMP},\text{GDPc}\;\right)$$

FPI captures Food Production Index, PS measures Political Stability, UNEMP stands for Unemployment and GDPc the Gross Domestic Product per capita.

#### 3.2.2 Empirical Procedure

The empirical analysis began with the Pesaran (2004) Cross‐sectional Dependence (CD) test to determine whether observations across countries were independent (Table A.3). Given the evidence of cross‐sectional dependence for all the variables, the analysis proceeded to examine whether the variables exhibited spatial dependence using the Global Moran's *I* statistic. The spatial relationships among countries were defined using a first‐order contiguity spatial weight matrix (Table A.4), whereby two countries were considered neighbors if they shared a common land border. The contiguity matrix was row‐standardized so that the weights assigned to neighboring countries summed to one, allowing the estimated spatial effects to be interpreted as average neighborhood influences.

Following the confirmation of spatial dependence, the optimal specification was selected using the Akaike Information Criterion (AIC) and the Bayesian Information Criterion (BIC), where lower values indicate a better balance between model fit and parsimony (Table A.5). The Spatial Durbin Model (SDM) records the lowest AIC (5411.774) and BIC (5478.693) values, indicating that it provides the best fit to the data.

The SDM is specified based on the theoretical framework as follows:

(6) $$\mathrm{FS}I_{\mathrm{it}}=\delta \mathrm{FS}I_{\mathrm{it}-1}+\rho \mathrm{WFS}I_{\mathrm{it}}+\beta X_{\mathrm{it}}+\mathrm{\theta W}X_{\mathrm{it}}+\mu_{i}+\lambda_{t}+\varepsilon_{\mathrm{it}}$$

W is the spatial weight matrix, ρ captures endogenous spatial interaction and θ captures exogenous spillovers.

Compared with alternative models, the SDM estimates both endogenous and exogenous spatial spillovers simultaneously and produces unbiased estimates even when the true process follows either the SAR or SEM model (Elhorst 2014; LeSage and Pace 2009). The SDM with spatial $\left(\mu_{i}\right)$ and time fixed effects $\left(\lambda_{t}\right)$ is estimated via the maximum likelihood (ML) method following the approach developed by Lee and Yu (2010). The dynamic specification incorporates both the temporal lag of the dependent variable $\left({\mathrm{FSI}}_{t-1}\right)$ and the spatial–temporal lag of the dependent variable $\left(W{\mathrm{FSI}}_{t-1}\right)$ using the $\text{dlag}\left(3\right)$ option.

To mitigate endogeneity biases arising from reverse causality and omitted variables, the model includes spatial and temporal fixed effects, which control for time‐invariant unobserved heterogeneity (e.g., agro‐ecological conditions) and common time shocks (e.g., global food price fluctuations). Furthermore, the ML estimator for dynamic spatial panel models has been shown to produce consistent and asymptotically normal parameter estimates when both the cross‐sectional dimension (N) and the time dimension (T) are moderately large. In addition, the bias‐correction procedure of Lee and Yu (2010) effectively addresses the incidental parameters problem inherent in dynamic fixed‐effects specifications. Post‐estimation, the spatial autocorrelation of residuals is assessed using Moran's *I* test to confirm that the model has successfully accounted for spatial dependence (Table A.6).

## 4 Results and Discussion

### 4.1 Spatial Patterns of Plant and Animal Species Loss in SSA

Figure 1 presents the spatial distribution of plant and animal species loss across SSA for 2003, 2013 and 2022. Collectively, the maps reveal a progressive increase in biodiversity loss over the two‐decade period. In 2003, both plant and animal species loss was relatively limited across most of SSA, with only a few countries exhibiting high levels of biodiversity decline. Plant species loss was mainly concentrated in parts of Central and Southern Africa, while animal species loss was largely confined to Southern Africa. By 2013, the spatial extent of biodiversity loss had expanded considerably. Plant species loss became more pronounced across Central and Eastern Africa, with several countries transitioning into higher loss categories. A similar pattern is observed for animal species, where countries in Eastern, Central and Southern Africa recorded increasing levels of wildlife decline. The 2022 maps demonstrate a continued deterioration in both plant and animal biodiversity. Darker shades became increasingly widespread, particularly across Central, Eastern and Southern Africa, reflecting higher levels of species loss than in previous years. While some West African countries also experienced increasing biodiversity decline. Comparatively, plant species loss appears to have spread more extensively across SSA, whereas animal species loss remained concentrated in biodiversity‐rich landscapes but exhibited greater intensity within those areas.

**FIGURE 1:**
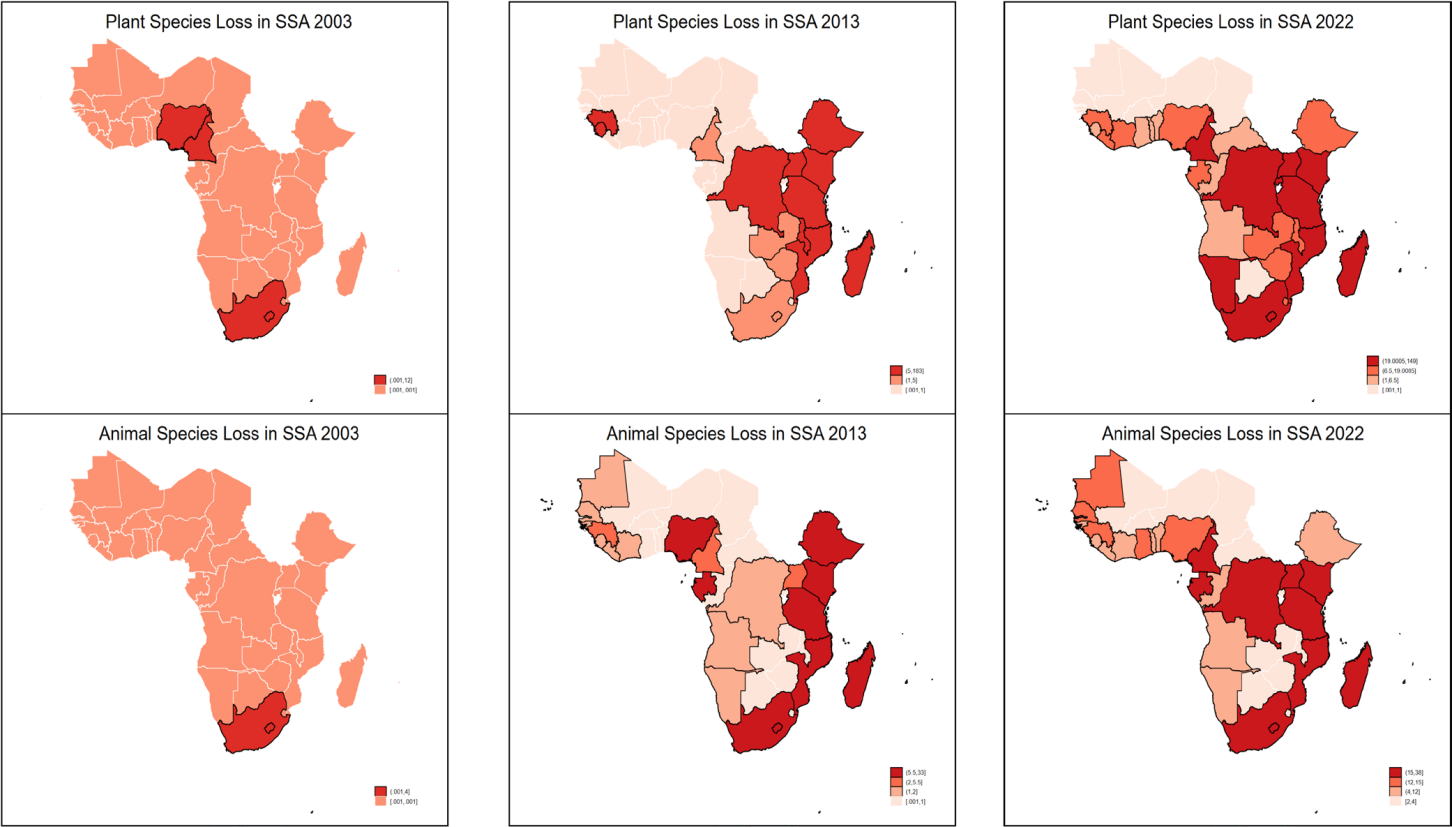
Spatial distribution of plant species loss in Sub‐Saharan Africa. *Source:* Author's construction 2026 using STATA 17.

The observed increase in both plant and animal species loss highlights the growing pressure on natural ecosystems in SSA. The expansion of agriculture, deforestation, urbanization, infrastructure development, and the increasing impacts of climate change have collectively contributed to habitat degradation and fragmentation, resulting in declining populations of both flora and fauna. The similarity in the spatial patterns of plant and animal species loss suggests that these pressures are simultaneously affecting multiple components of biodiversity rather than isolated taxonomic groups.

### 4.2 Descriptive Statistics

Table 2 presents the descriptive statistics, providing an overview of the central tendency, variability and distribution of the variables included in the empirical model. The FSI, which serves as the dependent variable, has a standardized mean of 0.000 and a standard deviation of 1.362. Its values range from −3.276 to 3.455, indicating considerable variation in food security outcomes across countries and over time. The standardization of the index facilitates comparisons across observations while preserving differences in relative food security performance. Biodiversity, measured using the RLI, has a mean value of 0.873 and a relatively small standard deviation of 0.096. The index ranges from 0.393 to 0.990, indicating that although biodiversity conditions differ across countries, the variation is less pronounced than that observed for the socioeconomic indicators. The Food Production Index reports a mean of 91.89 with a standard deviation of 22.49. Values range from 33.02 to 183.45, demonstrating considerable differences in agricultural production performance across the sample.

**TABLE 2:** Descriptive statistics.

Variable	Obs	Mean	SD	Min	Max
Food Security Index	880	0	1.362	−3.276	3.455
Red List Index	880	0.873	0.096	0.393	0.990
Food production index	880	91.887	22.486	33.02	183.450
Political Stability	880	−0.471	0.844	−2.699	1.686
ND‐GAIN Index	880	38.298	5.428	25.664	57.757
Unemployment	880	7.834	7.114	0.316	35.359
Population	880	21,700,247	32,540,302	146,375	2.232e+08
GDP per capita	880	1789.724	2077.500	109.594	11,643.460

*Source:* Authors estimation 2026 using STATA 17.

Political Stability exhibits an average score of −0.471, with values ranging from −2.699 to 1.686. The negative mean indicates that, on average, countries in the sample experience relatively weak political stability. The wide range further reflects substantial heterogeneity in governance and institutional environments across SSA. The ND‐GAIN Index, which measures vulnerability and readiness to adapt to climate change, has an average score of 38.30 and a standard deviation of 5.43. The index ranges from 25.66 to 57.76, suggesting moderate variation in countries' adaptive capacity. The relatively low average score indicates that many countries remain vulnerable to climate‐related risks that could adversely affect agricultural systems and food security. The unemployment rate averages 7.83%, with a standard deviation of 7.11 percentage points. The observed range, from 0.32% to 35.36%, points to substantial differences in labor market conditions across the sample. Such variation may influence household purchasing power and access to food, particularly in economies where wage employment constitutes an important source of income. Population displays the greatest variability, with an average of approximately 21.7 million people and a standard deviation exceeding 32.5 million. Population sizes range from approximately 146,000 to more than 223 million, reflecting the demographic diversity of Sub‐Saharan African countries. GDP per capita averages US$1789.72, accompanied by a relatively high standard deviation of US$2077.50. Values range from approximately US$109.59–US$11,643.46, indicating marked disparities in economic development across the sampled countries.

Overall, the descriptive statistics reveal substantial heterogeneity among Sub‐Saharan African countries in terms of food security, biodiversity, agricultural productivity, governance, climate resilience and socioeconomic conditions. To reduce skewness and limit the influence of extreme observations, highly skewed continuous variables were transformed using their natural logarithms prior to regression estimation. Descriptive statistics are reported in the original units.

### 4.3 Global Spatial Autocorrelation

Table 3 presents the results of the Global Moran's *I* test, which was conducted to determine whether the variables exhibit spatial dependence. The test evaluates the null hypothesis of spatial randomness, whereby observations are assumed to be randomly distributed across space. A statistically significant positive Moran's *I* statistic indicates positive spatial autocorrelation, implying that countries with similar characteristics tend to be geographically clustered. Conversely, an insignificant Moran's *I* statistic suggests the absence of systematic spatial dependence.

**TABLE 3:** Spatial autocorrelation analysis.

Variables	Moran's *I*	*E*(*I*)	SE(*I*)	*Z*(*I*)	*p*
Red List Index	0.312	−0.026	0.051	6.633	0.000***
Food Security Index	0.044	−0.026	0.041	1.668	0.095*
Unemployment	0.234	−0.026	0.054	4.784	0.000***
GDP per capita	0.153	−0.026	0.082	2.171	0.030**
ND‐GAIN Index	0.143	−0.026	0.085	1.984	0.047**
Political stability	0.047	−0.026	0.036	2.030	0.042**
Food production index	−0.076	−0.026	0.055	−0.900	0.368
Population	−0.016	−0.026	0.047	0.208	0.835

*Source:* Author’s estimation 2026 using STATA 17.

* *p* < 0.1.

** *p* < 0.05.

*** *p* < 0.01.

The findings reveal that most of the study variables exhibit statistically significant positive spatial autocorrelation. These results suggest that geographical proximity plays an important role in shaping biodiversity, food security and several socioeconomic conditions within the region. This pattern is expected given the shared climatic conditions, ecological systems, regional trade networks and institutional linkages among neighboring Sub‐Saharan African countries. The presence of significant spatial dependence therefore provides strong econometric justification for adopting a spatial econometric approach.

### 4.4 Red List Index and Food Security

Table 4 presents the Spatial Durbin Model (SDM) estimates of the relationship between biodiversity and food security in SSA. The model reports the main regression results together with the short‐run (direct, indirect and total) and long‐run effects while controlling for socioeconomic, institutional and environmental factors. The model explains a substantial proportion of the variation in food security (*R* ^2^ = 0.919), suggesting that the selected variables provide a good representation of the determinants of food security across the sampled countries.

**TABLE 4:** Effect of biodiversity on food security.

Variables	(1)	(2)	(3)	(4)	(5)	(6)	(7)	(8)
	Main	Wx	SR direct	SR indirect	SR total	LR direct	LR indirect	LR total
Lag of FSI	0.961***							
	(0.013)							
Lag of weighted FSI	−0.024							
	(0.176)							
Red List Index	0.053	−0.200	0.058	−0.101	−0.043	4.110	−3.423	0.688
	(0.083)	(0.434)	(0.080)	(0.211)	(0.219)	(59.877)	(40.233)	(59.231)
Unemployment	−0.133***	0.567**	−0.130***	0.259**	0.129	−5.325	2.587	−2.738
	(0.050)	(0.236)	(0.048)	(0.109)	(0.118)	(55.400)	(38.208)	(51.745)
Log of GDP per capita	0.188	2.008	0.197	0.997	1.194*	−4.211	8.969	4.758
	(0.363)	(1.239)	(0.345)	(0.621)	(0.688)	(142.287)	(221.596)	(207.236)
Food production index	0.020***	0.028	0.020***	0.013	0.034**	0.138	−0.055	0.083
	(0.006)	(0.030)	(0.006)	(0.014)	(0.016)	(5.737)	(5.648)	(3.725)
Log population	−7.109***	−6.380	−7.173***	−3.214	−10.386***	−203.422	50.113	−153.308
	(1.635)	(5.357)	(1.716)	(2.277)	(2.773)	(3056.727)	(1824.949)	(2533.693)
ND‐GAIN Index	−0.057	0.268	−0.058	0.118	0.059	−1.751	2.043	0.292
	(0.055)	(0.204)	(0.054)	(0.089)	(0.099)	(19.149)	(21.099)	(18.627)
Political stability	0.634***	−1.344*	0.646***	−0.604*	0.043	18.273	−12.231	6.042
	(0.206)	(0.752)	(0.199)	(0.362)	(0.374)	(233.990)	(143.338)	(146.503)
Spatial autoregressive parameter				0.078				
				(0.206)				
Variance of the idiosyncratic error				3.786***				
				(0.176)				
Spatial fixed‐effect	Yes	Yes	Yes	Yes	Yes	Yes	Yes	Yes
Time fixed‐effects	Yes	Yes	Yes	Yes	Yes	Yes	Yes	Yes
Observations	840	840	840	840	840	840	840	840
*R* ^2^	0.919	0.919	0.919	0.919	0.919	0.919	0.919	0.919
Number of id	40	40	40	40	40	40	40	40

*Note:* *W* is the spatial weights matrix. SR is the short run and LR is the long run effect. Standard errors in parentheses.

*Source:* Author’s estimation 2026 using STATA 17.

* *p* < 0.1.

** *p* < 0.05.

*** *p* < 0.01.

The estimated coefficient for the RLI is positive but statistically insignificant in both the short and long run, suggesting that biodiversity conservation does not exert a measurable effect on the aggregate FSI during the study period. However, this finding should be interpreted with caution and does not necessarily imply that biodiversity is unimportant for food security in SSA. Rather, it is likely to reflect the multidimensional nature of the dependent variable used in the study. The FSI employed in the analysis is a composite measure that combines several dimensions of food security, each capturing different aspects of food availability and nutritional adequacy, which may respond differently to changes in biodiversity. Consequently, aggregating them into a single index may obscure the specific pathways through which biodiversity influences food security. Accordingly, a disaggregated analysis of the FSI would provide a more nuanced understanding of the biodiversity–food security nexus.

Among the control variables, unemployment exerts a negative and statistically significant direct effect on food security (*β* = −0.130). The result indicates that higher unemployment reduces households' purchasing power and limits economic access to food, thereby worsening food security. Although the indirect spatial effect is positive and significant (*β* = 0.259), the overall short‐run total effect is statistically insignificant, suggesting that positive spillovers from neighboring countries are insufficient to offset domestic labor market constraints. This finding is consistent with the broader food security literature, which identifies employment and income as critical determinants of household food access (FAO, IFAD, UNICEF, WFP, WHO 2023). It also supports the Sustainable Livelihoods Framework, which emphasizes that financial capital is essential for securing food, particularly in developing economies where wage income constitutes an important source of household welfare.

GDP per capita exhibits a positive but statistically insignificant direct effect on food security, although the short‐run total effect is positive and significant at the 10% level. This suggests that improvements in income contribute to food security primarily through broader economy‐wide spillover effects rather than through direct domestic impacts. Similar findings have been reported in cross‐country analyses showing that economic growth improves food security only when accompanied by equitable income distribution, effective institutions and agricultural transformation (Timmer 2015).

The Food Production Index has a positive and statistically significant direct effect in both the main model (*β* = 0.020) and the short‐run direct effect (*β* = 0.020), while the short‐run total effect also remains positive and significant (*β* = 0.034). These findings indicate that improvements in agricultural production contribute directly to enhanced food security. The result aligns with extensive empirical evidence showing that increased agricultural productivity strengthens food availability, stabilizes food supplies and improves household welfare (FAO, IFAD, UNICEF, WFP, WHO 2023; Pretty 2008). Nevertheless, the insignificant long‐run spillover effects suggest that increases in domestic food production primarily benefit the producing country rather than generating substantial regional spillover effects.

Population displays a strong negative relationship with food security. The direct short‐run effect (*β* = −7.173) and the total short‐run effect (*β* = −10.386) are both statistically significant, indicating that rapid population growth places considerable pressure on food systems. This finding supports Malthusian perspectives (Malthus 1798), which argue that population growth can outpace food production when productivity improvements are insufficient. Although technological progress has mitigated these pressures globally, many countries in SSA continue to experience rapid demographic expansion alongside relatively slow agricultural transformation, thereby increasing pressure on land, biodiversity and food supplies.

Political stability emerges as another important determinant of food security. The positive and highly significant direct effect (*β* = 0.646) indicates that improvements in governance and political stability enhance food security by creating an enabling environment for agricultural investment, functioning markets and effective public service delivery. Studies such as Etienne et al. (2025) demonstrate that political stability has a statistically significant positive effect on cereal yields. Stable governance creates the conditions for long‐term investment in agriculture, which is critical for boosting production and ensuring food availability. However, the negative indirect effect (*β* = −0.604) suggests that political developments in neighboring countries may generate adverse spillover effects, possibly through migration pressures, trade disruptions, or regional conflicts. Abdullah et al. (2025) similarly found that a country's food security is significantly affected not only by its own political stability but also by that of its neighboring countries.

Finally, the ND‐GAIN Index does not exert a statistically significant influence on food security in either the short or long run. Although climate resilience is theoretically expected to improve agricultural performance, the insignificant coefficients suggest that adaptive capacity alone may be insufficient to enhance food security without complementary investments in agricultural technology, infrastructure and institutional quality. This finding is consistent with studies arguing that climate adaptation policies are most effective when integrated with broader development and food system strategies rather than implemented in isolation (Du et al. 2026).

Taken together, the results indicate that socioeconomic and institutional factors exert a stronger immediate influence on food security than biodiversity at the macroeconomic level.

The Global Moran's *I* test on the residuals, presented in Table A.6, yields a *p*‐value of 0.412, which exceeds the conventional significance levels (1%, 5% and 10%). This finding indicates that the residuals do not exhibit statistically significant spatial autocorrelation, implying that the Spatial Durbin Model has successfully accounted for the spatial dependence present in the data.

### 4.5 Disaggregated Analysis: Effect of Biodiversity on Food Security Components

To further investigate the insignificant relationship between biodiversity and the aggregate FSI, the analysis was extended by estimating separate Spatial Durbin Models for each component of the index (Table 5). Specifically, the FSI was disaggregated into Average Dietary Energy Supply Adequacy (ADESA), Share of Dietary Energy Supply Derived from Cereals, Roots and Tubers (SDES), Average Protein Supply (APS) and Per Capita Food Supply Variability (PCFSV). This approach allows the identification of the specific dimensions of food security that are more responsive to biodiversity conservation.

**TABLE 5:** Disaggregated analyses.

Variables	(1)	(2)	(3)	(4)	(5)	(6)	(7)	(8)
	Main	Wx	SR direct	SR indirect	SR total	LR direct	LR indirect	LR total
*Effect of the Red List Index on the average dietary energy supply adequacy (ADESA)*								
Lag of ADESA	0.973***							
	(0.011)							
Lag of the Weighted ADESA	0.422***							
	(0.157)							
Red List Index	0.096*	−0.328	0.105**	−0.143	−0.038	3.433	−6.860	−3.427
	(0.053)	(0.271)	(0.052)	(0.113)	(0.110)	(30.963)	(75.251)	(74.514)
Unemployment	−0.080**	0.745***	−0.089***	0.309***	0.219***	−5.718	7.311	1.593
	(0.033)	(0.152)	(0.032)	(0.062)	(0.065)	(68.309)	(111.761)	(106.741)
Log of GDP per capita	0.465**	0.934	0.453**	0.364	0.817**	0.044	9.727	9.771
	(0.235)	(0.782)	(0.227)	(0.345)	(0.367)	(280.676)	(325.592)	(284.412)
Food production index	0.012***	0.011	0.012***	0.003	0.015*	0.073	0.200	0.274
	(0.004)	(0.019)	(0.004)	(0.008)	(0.008)	(5.574)	(8.243)	(7.897)
Log of population	−5.716***	−6.657**	−5.676***	−2.132*	−7.808***	−28.263	−78.673	−106.936
	(1.072)	(3.217)	(1.125)	(1.266)	(1.520)	(2498.411)	(3336.214)	(3078.108)
ND‐GAIN Index	0.006	0.248*	0.001	0.097*	0.098*	−2.657	1.276	−1.381
	(0.036)	(0.132)	(0.035)	(0.050)	(0.054)	(42.836)	(34.093)	(18.504)
Political Stability	0.452***	−0.159	0.467***	−0.109	0.358*	0.523	−2.186	−1.662
	(0.129)	(0.483)	(0.128)	(0.201)	(0.193)	(224.744)	(177.667)	(92.143)
Spatial autoregressive parameter			0.241					
			(0.166)					
Variance of the idiosyncratic error			1.596***					
			(0.074)					
*Effect of the Red List Index on the Share of dietary energy supply derived from cereals, roots and tubers (SDES)*								
Lag of SDES	1.012***							
	(0.015)							
Lag of the Weighted SDES	−0.001							
	(0.115)							
Red List Index	0.070*	−0.083	0.073**	−0.034	0.039	−1.142	1.886	0.744
	(0.037)	(0.208)	(0.035)	(0.096)	(0.095)	(28.725)	(31.599)	(34.722)
Unemployment	−0.043*	0.171	−0.042*	0.078	0.036	−1.154	0.215	−0.940
	(0.023)	(0.110)	(0.023)	(0.049)	(0.054)	(51.293)	(27.871)	(45.371)
Log of GDP per capita	0.076	−0.617	0.073	−0.265	−0.192	0.051	−1.082	−1.032
	(0.167)	(0.534)	(0.160)	(0.262)	(0.296)	(87.792)	(122.492)	(140.849)
Food production index	0.001	−0.037***	0.001	−0.018***	−0.017**	−0.128	−0.087	−0.215
	(0.003)	(0.013)	(0.003)	(0.006)	(0.007)	(2.352)	(7.213)	(7.800)
Log of population	−0.377	6.126***	−0.361	2.843***	2.483**	15.002	27.040	42.042
	(0.756)	(2.057)	(0.776)	(1.006)	(1.242)	(311.508)	(1297.877)	(1379.931)
ND‐GAIN Index	0.032	−0.075	0.031	−0.035	−0.004	−0.187	1.954	1.767
	(0.025)	(0.092)	(0.024)	(0.042)	(0.047)	(23.528)	(29.073)	(34.380)
Political Stability	−0.158*	1.011***	−0.145	0.464***	0.320*	1.876	−1.477	0.400
	(0.092)	(0.341)	(0.089)	(0.162)	(0.166)	(100.457)	(123.169)	(139.205)
Spatial autoregressive parameter			0.095					
			(0.108)					
Variance of the idiosyncratic error			0.794***					
			(0.037)					
*Effect of the Red List Index on the Average protein supply (APS)*								
Lag of APS	0.995***							
	(0.013)							
Lag of the weighted APS	−0.085							
	(0.432)							
Red List Index	−0.001	0.115	−0.004	0.007	0.003	0.118	0.416	0.534
	(0.047)	(0.344)	(0.050)	(0.246)	(0.263)	(10.280)	(14.297)	(12.886)
Unemployment	−0.068**	0.039	−0.070**	0.008	−0.062	−1.863	0.781	−1.081
	(0.028)	(0.134)	(0.029)	(0.083)	(0.089)	(10.941)	(18.162)	(15.457)
Log of GDP per capita	−0.240	1.827***	−0.236	0.907**	0.671	−0.205	6.911	6.706
	(0.220)	(0.676)	(0.202)	(0.459)	(0.494)	(83.384)	(206.327)	(204.755)
Food production index	0.005	0.023	0.005	0.010	0.015	0.193	0.109	0.302
	(0.004)	(0.020)	(0.004)	(0.009)	(0.010)	(1.176)	(2.671)	(2.682)
Log of population	−2.595***	−3.884	−2.553**	−1.537	−4.090**	−61.195	−47.570	−108.764
	(0.925)	(5.065)	(1.014)	(1.874)	(2.038)	(608.533)	(954.328)	(819.664)
ND‐GAIN Index	−0.061*	0.297**	−0.062**	0.140**	0.078	−0.820	0.384	−0.436
	(0.032)	(0.117)	(0.031)	(0.066)	(0.074)	(14.853)	(23.169)	(20.427)
Political stability	0.419***	−1.343***	0.427***	−0.641**	−0.214	4.013	2.040	6.053
	(0.127)	(0.429)	(0.113)	(0.279)	(0.283)	(110.319)	(141.887)	(108.233)
Spatial autoregressive parameter			0.029					
			(0.512)					
Variance of the idiosyncratic error			1.208***					
			(0.056)					
*Effect of the Red List Index on the per capita food supply variability (PCSV)*								
L.PCFSV	0.876***							
	(0.022)							
L.WPCFSV	−0.126							
	(0.134)							
Red List Index	−0.228	−0.537	−0.170	−0.243	−0.414	−1.259	−2.593	−3.852
	(0.542)	(2.765)	(0.518)	(1.247)	(1.218)	(5.490)	(28.886)	(30.017)
Unemployment	0.398	−0.757	0.397	−0.341	0.056	3.842	−2.463	1.379
	(0.330)	(1.567)	(0.323)	(0.683)	(0.738)	(3.451)	(21.104)	(22.369)
Log of GDP per capita	0.728	7.603	0.733	3.712	4.444	3.891	24.656	28.547
	(2.408)	(8.296)	(2.302)	(3.909)	(4.340)	(22.286)	(90.231)	(97.391)
Food production index	−0.055	0.239	−0.052	0.103	0.050	−0.533	0.911	0.378
	(0.040)	(0.190)	(0.040)	(0.085)	(0.099)	(0.421)	(2.042)	(2.184)
Log of population	7.640	−33.735	7.331	−15.002	−7.671	75.030	−117.614	−42.584
	(10.868)	(29.679)	(11.302)	(14.283)	(17.428)	(106.264)	(362.031)	(382.796)
ND‐GAIN Index	0.011	1.812	0.004	0.799	0.804	−0.581	6.364	5.782
	(0.359)	(1.306)	(0.347)	(0.572)	(0.631)	(3.541)	(11.052)	(11.804)
Political stability	1.385	−6.429	1.499	−2.904	−1.404	15.948	−23.977	−8.029
	(1.333)	(4.956)	(1.313)	(2.259)	(2.252)	(15.135)	(38.015)	(39.581)
Spatial autoregressive parameter			0.017					
			(0.109)					
Variance of the idiosyncratic error			167.606***					
			(7.806)					
Spatial fixed‐effect	Yes	Yes	Yes	Yes	Yes	Yes	Yes	Yes
Time fixed‐effects	Yes	Yes	Yes	Yes	Yes	Yes	Yes	Yes

*Source:* Authors estimation 2026 using STATA 17.

* means significant at 10% *p* < 0.1.

** means significant at 5% *p* < 0.05.

*** means significant at 1% *p* < 0.01.

The results reveal considerable heterogeneity in the effect of biodiversity across the four dimensions of food security. Unlike the aggregate analysis, biodiversity exhibits a positive and statistically significant relationship with both ADESA and SDES, while remaining insignificant for APS and PCFSV.

For ADESA, the RLI is positive and statistically significant in both the main model (*β* = 0.096) and the short‐run direct effect (*β* = 0.105). These findings suggest that improved biodiversity contributes to greater dietary energy availability within countries. Biodiversity enhances ecosystem services that improve agricultural productivity and increase the availability of staple foods. However, neither the short‐run total effect nor any of the long‐run effects are statistically significant, indicating that these benefits are primarily domestic and do not generate measurable spatial spillover effects across neighboring countries.

A similar pattern is observed for SDES. The RLI has a positive and statistically significant coefficient in the main specification (*β* = 0.070) and in the short‐run direct effect (*β* = 0.073), while the indirect and total effects remain statistically insignificant. This result suggests that biodiversity contributes to the production of staple crops, which remain the principal source of dietary energy across SSA. Healthy ecosystems support the ecological processes that sustain cereal production through improved pollination, soil health, water regulation and natural pest control, thereby strengthening food availability.

Taken together, the disaggregated analysis provides important insights into the biodiversity–food security relationship. The insignificant effect observed for the aggregate FSI appears to result from the combination of several dimensions that respond differently to biodiversity. Positive effects on dietary energy adequacy and staple food availability are offset by insignificant effects on protein supply and food supply stability, leading to an overall insignificant coefficient in the aggregate model. This finding highlights the importance of recognizing the multidimensional nature of food security when evaluating the contribution of biodiversity.

The disaggregated results are broadly consistent with previous empirical evidence showing that biodiversity contributes to specific dimensions of food security rather than uniformly improving all aspects of the food system. Studies have demonstrated that biodiversity enhances agricultural productivity through ecosystem services, which primarily improve food availability and dietary energy supply (Dawson et al. 2019; Garibaldi et al. 2013; Wagg et al. 2014). Similarly, Lachat et al. (2018) found that greater biological diversity is associated with improved dietary adequacy. However, the absence of significant effects on average protein supply and per capita food supply variability suggests that these dimensions are more strongly influenced by socioeconomic factors, market conditions, infrastructure and trade than by biodiversity alone. These findings reinforce the argument that the contribution of biodiversity to food security is multidimensional and may be obscured when food security is measured using a composite index, thereby highlighting the importance of disaggregated analyses in identifying the specific pathways through which biodiversity supports food systems.

### 4.6 Sensitivity Analysis

To assess the robustness of the main findings, the FSI was replaced with the Prevalence of Undernourishment, one of the most widely used indicators of food insecurity. Unlike the composite FSI, the Prevalence of Undernourishment measures the proportion of the population whose habitual dietary energy consumption falls below the minimum energy requirement, thereby providing a direct measure of food deprivation.

The results presented in Table 6 indicate that the RLI has a statistically significant negative effect on the Prevalence of Undernourishment. The coefficient is negative and significant in the main model (*β* = −0.175), while the short‐run direct effect is also negative and highly significant (*β* = −0.337). Since undernourishment is an inverse measure of food security, these findings imply that improvements in biodiversity reduce the incidence of undernourishment, thereby enhancing food security. Furthermore, the positive and significant short‐run indirect effect (*β* = 0.967) indicates the presence of spatial spillover effects, suggesting that changes in biodiversity conditions in neighboring countries influence domestic undernourishment levels. The significant positive short‐run total effect (*β* = 0.630) reflects the combined influence of these direct and indirect effects. However, none of the long‐run effects are statistically significant, indicating that the influence of biodiversity on undernourishment is primarily a short‐run phenomenon.

**TABLE 6:** Effect of RLI on the prevalence of undernourishment.

Variables	(1)	(2)	(3)	(4)	(5)	(6)	(7)	(8)
	Main	Wx	SR direct	SR indirect	SR total	LR direct	LR indirect	LR total
Lag undernourishment	1.047***							
	(0.010)							
Lag weighted undernourishment	1.533***							
	(0.127)							
Red List Index	−0.175***	2.791***	−0.337***	0.967***	0.630***	−2.774	0.464	−2.310
	(0.053)	(0.265)	(0.064)	(0.091)	(0.079)	(302.312)	(247.192)	(56.811)
Unemployment	0.082**	−0.642***	0.123***	−0.237***	−0.114***	−0.149	0.235	0.087
	(0.032)	(0.152)	(0.035)	(0.050)	(0.044)	(89.884)	(72.930)	(17.400)
Log of GDP per capita	1.146***	−2.435***	1.351***	−1.176***	0.175	−9.340	1.701	−7.639
	(0.234)	(0.761)	(0.253)	(0.279)	(0.252)	(845.096)	(686.559)	(162.492)
Food production index	−0.036***	−0.353***	−0.018***	−0.102***	−0.120***	0.655	0.116	0.772
	(0.004)	(0.019)	(0.005)	(0.007)	(0.007)	(11.070)	(8.980)	(2.166)
Log population	15.129***	73.819***	11.886***	18.632***	30.518***	−139.642	−79.971	−219.613
	(1.063)	(3.049)	(1.251)	(1.257)	(1.482)	(2420.701)	(2074.707)	(362.889)
NG‐GAIN Index	0.041	−0.664***	0.080**	−0.231***	−0.150***	−0.657	1.020	0.363
	(0.035)	(0.129)	(0.038)	(0.044)	(0.038)	(87.099)	(70.176)	(17.234)
Political stability	−0.423***	4.524***	−0.693***	1.614***	0.921***	−3.470	0.760	−2.710
	(0.128)	(0.483)	(0.146)	(0.179)	(0.147)	(519.404)	(423.767)	(98.481)
Spatial autoregressive parameter: rho			1.099***					
			(0.133)					
Variance of the idiosyncratic error sigma2_e			1.569***					
			(0.074)					
Spatial fixed‐effect	Yes	Yes	Yes	Yes	Yes	Yes	Yes	Yes
Time fixed‐effects	Yes	Yes	Yes	Yes	Yes	Yes	Yes	Yes
Observations	840	840	840	840	840	840	840	840
*R* ^2^	0.818	0.818	0.818	0.818	0.818	0.818	0.818	0.818
Number of _ID	40	40	40	40	40	40	40	40

*Note:* W is the spatial weights matrix. SR is the short run and LR the long run effect. Standard errors in parentheses.

*Source:* Author’s estimation 2026 using STATA 17.

* *p* < 0.1.

** *p* < 0.05.

*** *p* < 0.01.

The robustness analysis provides important evidence regarding the stability of the study's findings. These results are consistent with the disaggregated analysis reported in Table 5, where biodiversity significantly improved ADESA but had no measurable effect on APS or PCFSV. Since undernourishment is fundamentally determined by insufficient dietary energy intake, the significant negative relationship between biodiversity and undernourishment is consistent with the observed positive effect on dietary energy adequacy. Together, these results suggest that biodiversity contributes primarily to improving food availability through enhanced ecosystem services rather than uniformly affecting all dimensions of food security.

The robustness analysis therefore strengthens the conclusion that biodiversity contributes to food security in SSA, although its effects become more apparent when food security is measured using indicators that directly capture food availability rather than broad composite indices encompassing multiple dimensions.

## 5 Conclusion and Policy Implication

This study examined the effect of biodiversity on food security in SSA using a Dynamic Spatial Durbin Model estimated on panel data for 40 countries over the period 2001–2022. By explicitly accounting for temporal persistence, spatial dependence and cross‐country spillover effects, the analysis provides new evidence on the biodiversity–food security nexus in one of the world's most food‐insecure and biodiversity‐rich regions. The empirical findings reveal that the RLI does not exert a statistically significant effect on the aggregate FSI. However, the disaggregated analysis demonstrates that this insignificant aggregate relationship conceals important heterogeneity across the different dimensions of food security. Specifically, biodiversity significantly improves Average Dietary Energy Supply Adequacy and the Share of Dietary Energy Supply Derived from Cereals, Roots and Tubers, while no statistically significant effects are observed for Average Protein Supply or Per Capita Food Supply Variability. These results suggest that biodiversity primarily contributes to food availability through ecosystem services that enhance agricultural production, but its influence does not extend uniformly across all dimensions of food security. Consequently, the use of composite food security indicators may obscure important relationships that become apparent only when food security is analyzed through its constituent components. The robustness analysis using the Prevalence of Undernourishment further reinforces this conclusion by showing that improvements in biodiversity reduce undernourishment, confirming that biodiversity contributes to nutritional outcomes even when its effect is not evident in aggregate food security measures. The analysis also identifies several socioeconomic determinants of food security. Higher food production, political stability and income contribute positively to food security, whereas unemployment and rapid population growth undermine food security outcomes. These findings reaffirm that food security in SSA depends not only on ecological conditions but also on broader economic, demographic and institutional factors. Moreover, the significant spatial dependence identified in the data highlights the importance of regional interconnections, suggesting that food security and biodiversity conservation cannot be effectively addressed through national policies alone.

The findings have several important policy implications. First, the heterogeneous effects observed across the different dimensions of food security suggest that policymakers should avoid relying exclusively on composite food security indicators when designing interventions. Second, because biodiversity and ecosystem services extend beyond political boundaries, regional cooperation should be strengthened. Many ecosystems, river basins, forests, wildlife corridors and pollinator habitats are shared across countries in SSA. Regional institutions should therefore promote coordinated biodiversity conservation, transboundary ecosystem management and regional strategies for sustainable agriculture to maximize the benefits of shared natural capital. Third, biodiversity conservation alone cannot guarantee food security. The significant roles of agricultural production, employment, political stability and demographic pressures indicate that conservation policies should be complemented by broader investments in agricultural innovation, rural infrastructure, employment creation, governance and climate adaptation. Finally, the study highlights the need for future research to move beyond aggregate measures of food security. Future studies could investigate how biodiversity affects specific nutritional outcomes, food accessibility and dietary diversity using household‐level or subnational data. They could also distinguish between different dimensions of biodiversity, including plant, animal and ecosystem diversity, to better understand the ecological mechanisms through which biodiversity supports food security. Such analyses would provide more targeted evidence for designing policies that simultaneously promote biodiversity conservation and sustainable food systems.

## Funding

The authors have nothing to report.

## Ethics Statement

The authors have nothing to report.

## Consent

All authors have read and approved the final manuscript and consent to its publication in this journal.

## Conflicts of Interest

The authors declare no conflicts of interest.

## Supporting information


**Data S1:** Supporting Information.

## Data Availability

The data used in this study are derived from publicly available sources, including FAOSTAT at https://www.fao.org/faostat/en/#data/FS; the IUCN Red List of Threatened Species at https://www.iucnredlist.org; and World Bank databases at https://databank.worldbank.org/source/world‐development‐indicators. The final analytical dataset supporting the findings is provided as Supporting Information.

## Appendix A

**TABLE A.1:** Constructing the Food Security Index.

Component	Eigenvalue	Difference	Proportion	Cumulative
Component 1	1.855	0.754	0.464	0.464
Component 2	1.100	0.317	0.275	0.739
Component 3	0.783	0.521	0.196	0.935
Component 4	0.262		0.065	1.000

*Source:* Author's estimation 2026 using STATA 17.

**TABLE A.2:** Kaiser–Meyer–Olkin measure of sampling adequacy.

Variable	KMO
ADESA	0.471
SDES	0.390
APS	0.467
PCSV	0.336
Overall	0.448

*Source:* Author's estimation 2026 using STATA 17.

**TABLE A.3:** Cross‐sectional dependence test.

Variable	CD‐test	*p*	Average joint *T*	Mean *ρ*	Mean abs(*ρ*)
Food Security Index	44.918	0.000	22.00	0.34	0.59
Red List Index	40.421	0.000	22.00	0.31	0.82
Unemployment	11.442	0.000	22.00	0.09	0.45
Food production index	71.459	0.000	22.00	0.55	0.75
Political stability	4.895	0.000	22.00	0.04	0.43
ND‐GAIN Index	49.218	0.000	22.00	0.38	0.53
Population	128.86	0.000	22.00	0.98	0.98
GDP per capita	102.506	0.000	22.00	0.78	0.78

*Source:* Author's estimation 2026 using STATA 17.

**TABLE A.4:** Spatial‐weighting matrix W.

Matrix	Description
Dimensions	40×40
Stored as	40×40
Links	
Total	142
Min	0
Mean	3.55
Max	8

*Note:* Spatial‐weighting matrix contains 5 islands.

*Source:* Author's estimation 2026 using STATA 17.

**TABLE A.5:** Akaike's information criterion and Bayesian information criterion.

Model	*N*	AIC	BIC
Spatial autoregressive (SAR) model	880	5512.729	5555.748
**Spatial Durbin model (SDM)**	**880**	**5411.774**	**5478.693**
Spatial error model (SEM)	880	5513.278	5556.297
Spatial autocorrelation (SAC) model	880	5514.476	5562.275
Generalized spatial panel random‐effects (GSPRE) model	880	5774.888	5832.248

*Note:* Bold means that the spatial Durbin model is the appropriate model, the model with the lowest AIC and BIC.

*Source:* Author's estimation 2026 using STATA 17.

**TABLE A.6:** Moran's *I* test on residuals.

Variables	Moran's *I*	*E*(*I*)	SE(*I*)	*Z*(*I*)	*p*
Residuals	0.0206	−0.026	0.056	0.821	0.412

*Source:* Author's estimation 2026 using STATA 17.
